# Role of cGAS–Sting Signaling in Alzheimer’s Disease

**DOI:** 10.3390/ijms24098151

**Published:** 2023-05-02

**Authors:** Manoj Govindarajulu, Sindhu Ramesh, McNeil Beasley, Graham Lynn, Caleigh Wallace, Sammie Labeau, Suhrud Pathak, Rishi Nadar, Timothy Moore, Muralikrishnan Dhanasekaran

**Affiliations:** 1 Department of Drug Discovery and Development, Harrison College of Pharmacy, Auburn University, Auburn, AL 36849, USA; 2 Units Administration, Research Programs, Drug Discovery and Development, Harrison College of Pharmacy, Auburn University, 2316 Walker Building, Auburn, AL 36849, USA

**Keywords:** cGAS, STING, neuroinflammation, Alzheimer’s disease, type-I IFN

## Abstract

There is mounting evidence that the development of Alzheimer’s disease (AD) interacts extensively with immunological processes in the brain and extends beyond the neuronal compartment. Accumulation of misfolded proteins can activate an innate immune response that releases inflammatory mediators and increases the severity and course of the disease. It is widely known that type-I interferon-driven neuroinflammation in the central nervous system (CNS) accelerates the development of numerous acute and chronic CNS diseases. It is becoming better understood how the cyclic GMP–AMP synthase (cGAS) and its adaptor protein Stimulator of Interferon Genes (STING) triggers type-I IFN-mediated neuroinflammation. We discuss the principal elements of the cGAS–STING signaling pathway and the mechanisms underlying the association between cGAS–STING activity and various AD pathologies. The current understanding of beneficial and harmful cGAS–STING activity in AD and the current treatment pathways being explored will be discussed in this review. The cGAS–STING regulation offers a novel therapeutic opportunity to modulate inflammation in the CNS because it is an upstream regulator of type-I IFNs

## 1. Introduction

A key component of the immune defense system in many species is the ability to recognize foreign DNA. The foreign (viruses, bacteria, parasites) or self-DNA are sensed by several nucleic acid sensors, which elicit an immune response by activating several signaling cascades. Some of the well-studied DNA sensors include Toll-like receptor 9 (TLR9) and cyclic guanosine monophosphate (GMP)–adenosine monophosphate (AMP) synthase (cGAS), which is absent in melanoma 2 (AIM2) [1]. Normally, the DNA in eukaryotic cells is either confined to the mitochondria or nucleus. The nucleases in the cytosol and the endolysosomal compartments rapidly degrade any extracellular, mitochondrial or nuclear DNA that gains access to the cytosol [2]. Hence, the presence of excess foreign or self-DNA in the cytosol serves as a danger-associated molecular pattern (DAMP) and induces a strong type I interferon (IFN) response [3]. In the mammalian cells, the activation of cGAS and its downstream stimulator of interferon genes (STING) pathway leads to the generation of type I IFNs and pro-inflammatory cytokines and has recently emerged as a crucial mechanism for mediating DNA-mediated innate immune responses.

In comparison to the classical pattern-recognition receptors (PRRs) associated with pathogen-specific patterns, the cGAS system is ineffective in differentiating self-DNA from foreign DNA [4,5]. Hence, extracellular, mitochondrial and nuclear DNA are capable of activating cGAS when they gain access into the cytosol. However, multiple regulatory safety mechanisms are present to prevent the aberrant activation of cGAS-induced autoreactivity. Some of the mechanisms include (i) compartmentalization of cGAS to the cytosol and inner plasma membrane, (ii) tethering the cGAS in the nucleus to histones and keeping it in an inactive state despite the presence of genomic DNA, (iii) degradation of immunogenic DNA and cGAMP by nucleases and phosphodiesterases and (iv) sophisticated protein–protein interaction and post-translational modification (PTM) networks to maintain the stability and functionality of cGAS and STING [6]. Hence, dysregulation of these regulatory mechanisms leads to aberrant activation of the cGAS–STING pathway, which induces exaggerated immune responses that lead to sterile inflammatory conditions, including cardiometabolic disorders, neurodegenerative diseases, autoimmune diseases and cancers. Recent studies have shown that cGAS–STING signaling is involved in several neurodegenerative diseases, including Alzheimer’s. In this review, we briefly summarize cGAS–STING signaling and discuss the molecular mechanisms that cause aberrant activation of this pathway in AD. Furthermore, we discuss possible intervention strategies, their therapeutic implications for AD and their significance for clinical translation.

## 2. cGAS–STING Pathway

The cyclic GMP–ATP Synthase (cGAS) is a ~522 amino acid-long protein that contains a basic domain of approximately 160 unstructured base pairs at the N terminal and a globular ~360 amino acids at the C-terminal domain containing a nucleotidyltransferase [7] core domain (160–330) and the male abnormal 21 (Mab21) domain (213–513). The NTase core domain regulates the enzyme activity of cGAS, whereas the Mab21 domain regulates the specificity of cGAS toward dsDNA [8]. The cGAS is primarily a cytoplasmic protein that binds to DNA substrates, which include exogenous sources such as retrovirus, DNA viruses, and intracellular bacteria and endogenous sources such as dysfunctional mitochondria that release mitochondrial DNA (mtDNA) and chromatin fragments into the cytosol and micronuclei following mitotic or replicative crises and DNA damage [9]. cGAS is constitutively present as an inactive protein in the cell. On sensing the DNA substrates, the NTase domain of cGAS binds to the sugar–phosphate backbone of DNA, which induces a conformational change in the cGAS protein. This active cGAS interacts with ATP and GTP to produce a second messenger cyclic GMP–AMP (cGAMP).

The cGAMP is sensed by the endoplasmic reticulum (ER) membrane protein STING, which undergoes dimerization on binding to cGAMP. The activated STING translocates from the ER to the golgi compartment, and this trafficking is facilitated predominantly by coatomer protein complex II (COPII) vesicles [10]. Interestingly, the movement of STING protein out of the ER also promotes the formation of LC3 autophagic vesicles, the significance of which is discussed later. Once the STING reaches the golgi compartment, it recruits TANK- binding kinase 1 (TBK1) protein and promotes TBK1 autophosphorylation [11]. The activated TBK1, in turn, phosphorylates STING at Ser366 and the STING–TBK1 complex subsequently recruits the interferon regulatory factor 3 (IRF3) transcription factor [12]. The TBK1 also phosphorylates IRF3, induces dimerization and nuclear translocation and triggers the production of type I interferons and genes that encode inflammatory cytokines [13]. Another important signaling mediated by STING includes NF-κB-mediated transcriptional activation. Furthermore, cGAS–STING activation induces non-canonical NF-κB responses through the triggering of p52–RELB nuclear translocation. The activation of non-canonical NF-κB limits TBK1-mediated type I IFNs and the canonical NF-κB pathway; hence, it is a crucial negative regulator of STING effector mechanisms. Recent studies demonstrate that cGAS–STING signaling can modulate p53, MAPK p38 and STAT3 signaling, indicating additional potential pathways by which STING can affect cell states and cytokine output [9,14,15].

Mounting evidence indicates that autophagy plays an important role in regulating cGAS–STING signaling to maintain immune homeostasis. Several lines of evidence indicate that several ATG proteins are involved. Specifically, the binding of cGAS with Beclin-1 (an autophagy protein) promotes the autophagic degradation of both cytosolic DNA and cGAS itself [16]. Furthermore, the cGAS–Beclin-1 interaction suppresses cGAMP synthesis thereby preventing IFN production. This is due to the release of Rubicon, a negative autophagy regulator from the Beclin-1 complex, which induces autophagy. This regulatory process can be inhibited by type 1 IFN, which induces the E3 ligase TRIM14 to stabilize cGAS and prevent its degradation [17].

Similar to cGAS, STING induces autophagy. It does so through both canonical autophagy, involving the ULK complex and TBK1, and non-canonical autophagy, involving selective autophagy components (PI3P effector WIPI2 and the ATG5-12-16L1 complex) [18,19,20]. For instance, upon binding to cGAMP, the oligomerized STING migrates from the endoplasmic reticulum to the golgi via the ER–golgi intermediate compartment (ERGIC). At the ERGIC, STING is linked with the initiation of autophagy induction. STING-containing ERGIC serves as a membrane source of LC3 lipidation and subsequently induces autophagosome formation. Finally, the autophagosome fuses with the lysosome and degrades the content [10].

Furthermore, the LC-3-interacting regions of STING interact with LC3 to induce ATG5-dependent non-canonical autophagy. The LC3 lipidation induced by STING depends on WIP12 and ATG5 but not on ULK and VPS34–Beclin kinase complexes—the most important for autophagic signaling. Additionally, autophagy can degrade STING and prevent immune damage by involving TBK1 and p62 [21].

Importantly, autophagy induction by STING prevents viral replication/propagation and serves as a major host defense mechanism. Additionally, STING autophagy machinery prevents tumor cell overgrowth by engaging the cells in autophagic cell death [22]. Conversely, STING itself also becomes a target for degradation, which prevents overactivation. The autophagic components, in turn, regulate the STING activity in a feedback mechanism by mediating STING intracellular trafficking and lysosomal degradation. As a result, type 1 IFN production is increased in cells deficient in autophagy proteins or treated with drugs that prevent lysosomal acidification [23]. Additionally, autophagy also prevents STING activation by delivering cytosolic DNA to the lysosome, where it is degraded by DNase II [10]. Hence, autophagy is a key downstream event of STING that modulates to maintain homeostasis in a context-dependent way. In the context of increased accumulation of cytosolic DNA due to high cellular stress and impairment in autophagy, there can be a chronic unrestrained activation of the cGAS–STING pathway leading to inflammation. Furthermore, chronic activation leads to failure in compensatory responses, which further exacerbates autophagic dysfunction, leading to cell death.

The other important downstream signaling of cGAS–STING activation includes mediating cellular senescence and cell death. cGAS is an important inducer of the senescence-associated secretory phenotype (SASP) in response to DNA damage, particularly through the production of type 1 IFNs [24]. In an in vitro cell culture model, cGAS deficiency was shown to prevent the expression of senescence-associated inflammatory genes in response to DNA-damaging agents. Furthermore, STING-deficient mice also exhibited a reduced SASP phenotype [24], indicating that cGAS–STING mediates cellular senescence and prevents immortalization. Furthermore, activation of STING promotes the induction of cell-cycle inhibitors (p21) and proapoptotic BH3-only proteins [25,26]. The phosphorylated IRF3, downstream of STING activation, can also interact with proapoptotic proteins BAX and BAK and thereby lead to transcription-independent induction of apoptosis [4,27].

## 3. Activation of cGAS: Sources and Mechanisms

The primary mechanisms by which cGAS activation occurs are either due to dysfunctional DNA compartmentalization or its metabolism [28]. The different sources of DNA that drive the activation of cGAS include mitochondrial DNA (mtDNA). Its release into the cytosol is a key trigger for activating the cGAS–STING pathway. Mitochondrial DNA is a type of small, double-stranded circular molecule encased in the outer and inner mitochondrial membranes and has thousands of copies presenting in each cell (West and Shadel, 2017). Mitochondrial DNA is packed into nucleoids (unlike histones in the nuclear DNA) to counteract stress, and this process is regulated by mtDNA-binding protein, transcription factor A mitochondrial (TFAM). Aberrant mtDNA packing into nucleoids due to TFAM deficiency promotes mtDNA leakage into the cytosol and activation of the cGAS–STING pathway [29].

**Figure 1:**
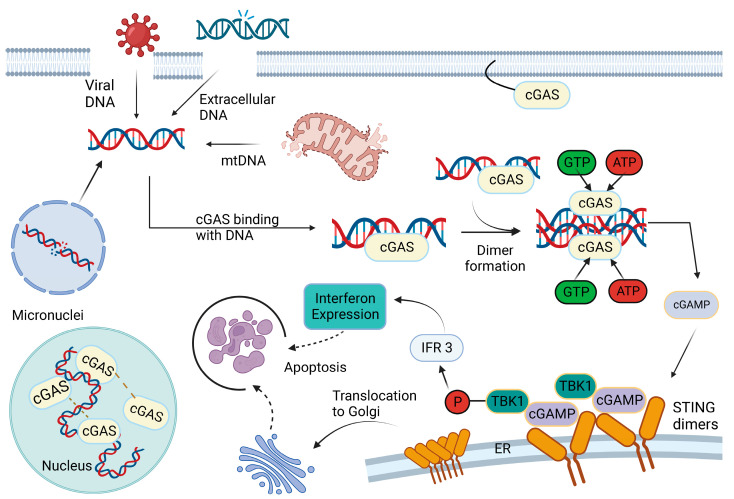
A schematic representation of double-stranded DNA (dsDNA)-induced activation of cytosolic cyclic GMP–AMP synthase (cGAS). Viral infection, mitochondrial damage, micronuclei or cellular stress leads to increased accumulation of cytoplasmic dsDNA, which binds to cGAS. On binding dsDNA, cGAS dimers assemble on dsDNA leading to enzymatic activation of cGAS and synthesis of 2′3′ cyclic GMP–AMP (cGAMP). The cGAMP binds to stimulator of interferon genes (STING) dimers present at the endoplasmic reticulum (ER) membrane, leading to profound conformational changes that trigger STING activation. STING recruits TANK-binding kinase 1 (TBK1), promoting TBK1 autophosphorylation and phosphorylation of interferon regulatory factor 3 (IRF3). Phosphorylated IRF3 translocates to the nucleus to induce gene expression of type I interferons and several other inflammatory mediators, pro-apoptotic genes and chemokines.

Secondly, the loss of mitochondrial membrane integrity due to mitochondrial dysfunction also allows mtDNA to leak into the cytosol (as seen in Figure 1). mtDNA can leak through the inner mitochondrial membrane and then be released through BAX/BAK macropores or by the formation of a mitochondrial permeability transition pore (MPTP). Alternatively, mtDNA can be released via voltage-dependent anion channel (VDAC) pores in the outer mitochondrial membrane. Third, mtDNA release can also be triggered by mitochondrial stress or damage. For instance, increased stress induced by a high-fat diet or fat-specific disulfide bond A oxidoreductase-like protein (DsbA-L) knockout promotes mtDNA release into the cytosol and activation of the cGAS–STING pathway, thereby leading to adipose tissue inflammation and insulin resistance [30,31]. Additionally, mtDNA stress induced by a lack of mitochondrial endonuclease G has been shown to activate cGAS [32]. Oxidative modification of mtDNA by mitochondrial reactive oxygen species renders mtDNA more resistant to processing by 3’ repair exonuclease 1 (TREX1) hence causing cGAS activation [33]. Defects in mitophagy machinery, wherein clearance of damaged mitochondria is diminished, are associated with increased STING activity. Absence of Parkin or PINK1 leads to a STING-mediated type I IFN response, which promotes the progression of neurodegeneration or systemic autoimmunity [34,35]. Hence, mitophagy limits inflammation by clearing damaged mitochondria to prevent cytosolic mtDNA accumulation. Overall, these studies suggest that cGAS is the primary sensor of mtDNA stress, and subsequent signaling promotes the progression of various sterile inflammatory diseases. Further in-depth research is required to determine whether targeting this pathway represents an effective therapeutic strategy for these diseases.

Extracellular self-DNA: Increased extracellular release of DNA from apoptotic and non-apoptotic cell death (necrosis, NETosis) has been linked to the activation of cGAS–STING. Under normal conditions, the released extracellular DNA is digested by deoxyribonuclease 1 (DNase1) and its homolog DNase1L3 in serum or degraded by DNase2 in the phagolysosomal compartment when engulfed by macrophages [36]. However, under certain disease conditions where there is an excess of ineffectively digested extracellular DNA, cGAS–STING activation ensues. For instance, in a myocardial infarction occlusion of the coronary arteries induces massive cardiac cell death, releasing self-DNA. This promotes the recruitment and activation of monocytes that trigger an interferon response in infiltrating leukocytes via the cGAS–STING-IRF3 pathway [37]. Similarly, in an aortic aneurysm and pancreatitis, DNA released from damaged aortic smooth muscle cells or pancreatic acinar cells activates cGAS–STING signaling in macrophages, thereby worsening the disease [38]. In sterile inflammatory diseases, neutrophil extracellular traps (NETs), a cell death process leads to the extrusion of neutrophil DNA–protein complexes into the extracellular space and, in turn, activates STING [39]. For instance, NET-induced cGAS–STING signaling also contributes to blood–brain barrier disruption in ischemic stroke after thrombolysis with tissue plasminogen activator therapy [40]. Finally, ionizing radiation-mediated tumor regression depends on the cGAS–STING-dependent cytosolic DNA sensing pathway through the internalization of tumor-cell-derived DNA in dendritic cells, which drives the adaptive immune response to radiation [41]. Impaired turnover of apoptotic cells can induce cGAS–STING activity. For instance, a deficiency in DNase II, an enzyme responsible for digesting the chromosomal DNA of apoptotic cells and nuclei, leads to inflammatory responses [42]. Consistently, genetic ablation of DNase II in mice leads to embryonic death because of the overactivation of STING signaling [43]. Finally, dying, or apoptotic cells are engulfed by phagocytic cells and targeted to lysosomes via LC3-associated phagocytosis (LAP). Hence, deficiency in LAP in the non-canonical form of autophagy promotes STING activation. Hence, these apoptotic pathways promote the sterile degradation of extracellular DNA, and if any of these pathways is compromised, cGAS–STING activation ensues. Since extracellular DNA plays a crucial role in many inflammatory diseases, there needs to be a better understanding of the mechanisms.

Cell-intrinsic genomic DNA: Recent studies indicate that cGAS is present in the nucleus; however, several inhibitory mechanisms restrict its catalytic activity on chromatin. For instance, the presence of barrier-to-autointegration factor (BAF) displaces cGAS from genomic DNA [44], thereby preventing cGAS activation in the nucleus. Suppression of BAF1 increases the accumulation of cGAS within the nucleus and induces a robust IFN response, as noted in the pathogenesis of autoimmune diseases [44]. Secondly, the sequestration of cGAS to chromatin (histone H2A and H2B in the nucleosomes) in the nucleus prevents cGAS interaction with self-genomic DNA (4). Hence, mutations of these key residues in the cGAS–histone binding site prevent nucleosome binding and promote nuclear DNA-dependent cGAS activation [45,46,47,48]. Alternatively, genomic DNA can leak into the cytosol and become accessible to cytosolic cGAS through several mechanisms. DNA damage caused by anticancer drugs induces nuclear DNA leakage into the cytosol and activates STING-dependent inflammation [49]. Defective DNA damage repair, as seen in ataxia-telangiectasia, leads to the accumulation of cytoplasmic fragments of genomic DNA, activates STING signaling in microglia and promotes neurodegeneration [50]. Nuclear DNA can become accessible to cytosolic cGAS through transcriptional derepression of LINE-1, as seen during aging, and cause an increase in cytoplasmic L1 cDNA, which initiates age-associated inflammation by activating the cGAS–STING pathway [51]. Additionally, the accumulation of L1 elements can occur due to the loss of 3 repair exonuclease TREX1, which normally functions to cleave the DNA intermediates.

DNA damage and subsequent cell-cycle progression through mitosis lead to the formation of micronuclei, which act as a repository for cGAS [52]. These micronuclei, which are made up of chromatin enclosed by their own nuclear membrane, occur due to the mis-segregation of DNA during cell division. The disintegration of the micronuclear membrane leads to cytosolic cGAS binding to damaged chromatin, leading to activation of the pro-inflammatory response [53]. Moreover, in senescent cells, the loss of lamin B1 with associated nuclear envelope disintegration leads to enveloped chromatin fragments budding off from the main nucleus [54]. These cytosolic chromatin fragments drive the production of the senescence-associated secretory phenotype by activating the cGAS–STING pathway, which is critical for the senescence-associated effects on aging and age-related disorders [25,55]. Defects in genes regulating de novo histone transcription that perturbs linker histone H1 expression can trigger abnormalities of cGAS localization and cGAS–STING-dependent type I IFN production [56]. Hence, coordinated regulation of cGAS within the nucleus is important to mediate normal homeostasis, and abnormal activation of cGAS leads to exacerbation of inflammation-associated diseases.

## 4. Role of cGAS–STING Pathway in Alzheimer’s Disease

One of the key pathophysiological mechanisms implicated in AD is mitochondrial dysfunction, characterized by mitochondrial damage and dysfunctional mitophagy caused by cellular stress due to the accumulation of toxic protein aggregates such as amyloid-β or hyperphosphorylated tau [57,58,59]. These proteins induce oxidative damage of mtDNA and induce double-stranded breaks in DNA [60]. Hence, the release of mtDNA and dsDNA fragments into the cytosol can act as a ligand for cGAS, which further activates STING and promotes a neuroinflammatory response [61]. Interestingly, the cGAS is negatively regulated by apoptosis in that the release of DNA from apoptotic cells does not activate the cGAS–STING pathway. However, it is activated by DNA released from intact cells undergoing cellular stress [62].

A study by Xie et al., demonstrated increased levels of cytosolic mtDNA in a 5xFAD mouse brain compared to aged-matched wild-type mice. Furthermore, increased cGAS–dsDNA interactions were demonstrated in human AD brains and the 5xFAD mouse model of AD. Increased phosphorylation of STING, TBK1, p-65 and IRF3, along with an increased type I IFN response, were noted in the prefrontal cortex of a human with AD and in aged wild-type mice samples. Genetic deletion of the cGAS gene in 5xFAD mice protected against cognitive impairment and ameliorated Aβ pathology and neuroinflammation. Furthermore, cGAS deficiency in microglia inhibited a neurotoxic A1 astrocytic phenotype, thus alleviating oligomeric amyloid-β peptide-induced neurotoxicity. Pharmacological inhibition of the cGAS–STING pathway using H-151, a STING inhibitor, effectively decreased protein phosphorylation (STING, TBK1, p-65 and IRF3), neuroinflammatory genes (*Tnfa*, *C1q*, *Il1a*, and *C3*) and decreased Aβ42 fractions in cortical tissues of 5xFAD mice [63].

In post-mortem human AD brain samples, increased STING expression was noted predominantly in CNS microvasculature and neuronal cells compared to age-matched control brain samples. The increased STING levels in neuronal cells were in close proximity to the amyloid plaques, and this colocalization of Aβ near STING expression may be due to the endoplasmic reticulum and mitochondrial stress on the cells caused by toxic protein accumulation, as previously indicated. Interestingly, STING expression was less evident in GFAP-positive astrocytes and CD68-positive microglia in the AD tissue [64]. Similarly, in a 5xFAD mouse model of AD, the activation of STING–IFN signaling following cGAS–dsDNA interaction was present mainly in the microglia but not in the neurons or astrocytes [63]. A study by Hou et al., demonstrated increased expression of cGAS and STING proteins in the brains of APP/PS1 mice in comparison to wild-type mice. Furthermore, treatment of HMC3 human microglial cells with the STING inhibitor H-151 prevented Aβ42-induced IL-6 production. Since the downstream signaling of STING activation involves a type I IFN response, and elevated levels of type I IFNs have been found in post-mortem human AD brains [65], the activation of STING could be linked to AD progression.

Neuroinflammation is considered to be a critical component in the pathogenesis of AD [66]. Accumulation of Aβ plaques, neurofibrillary tangles and degenerating neurons are classical stimulators of AD neuroinflammation [67,68]. Several lines of evidence indicate that cGAS and STING are predominantly expressed in the microglia, and the proinflammatory responses induced by activation of the cGAS–STING pathway are potent in microglia but less so in neurons and astrocytes [69,70]. In line with this, in a mouse model of chronic neurodegeneration, the upregulation of cytosolic DNA sensors, including cGAS, were found to drive type 1 IFN and pro-inflammatory cytokine production in microglia [71]. Similarly, the microglial cGAS–STING pathway has been shown to play a crucial role in mediating inflammation due to tau pathology [72]. In the 5xFAD mouse model of AD, the expression of phosphorylated STING co-localized with activated microglial marker CD68 around Aβ plaques suggested that the STING–IFN response occurs mainly in the microglia [63]. In a high-fat diet obese mouse model for prediabetes and cognitive impairment, increased expression of cGAS and STING was noted in the hippocampus as early as four days following high-fat diet feeding. The acute high-fat diet (HFD) also showed microglial activation and an increase in pro-inflammatory responses without any changes in cognition, indicating that the HFD promotes an acute and early pro-inflammatory response in the CNS that precedes or initiates several signaling cascades leading to neurodegeneration and cognitive impairment with chronic HFD [73]. Since inflammatory microglia play a critical role in AD pathogenesis and related neuroinflammation [74,75], the activation of cGAS–STING and its type I IFN respons, which releases various cytokines, can structurally and functionally injure the neurons [76].

The inflammatory response is one of the hallmarks of cellular senescence and SASPs are documented in various mouse models of AD [77,78]. As described earlier, the cGAS–STING pathway is linked to cellular senescence and inflammation. In a mouse model of AD (APP/PS1), cGAS expression was increased in 7-, 12- and 20-month-old AD mice in comparison to wild type. Furthermore, STING expression was higher in AD mice only after 12 months. The differences in cGAS and STING activation at different age groups could be attributed to different levels of immune activation at different ages. Furthermore, markers of cellular senescence (SA-β-gal staining and p16INK4a) increased in the hippocampus and cortex of AD mice. A microarray analysis on the hippocampus and cortex from 12-month-old APP/PS1 AD mice showed several immune and inflammatory response genes to be upregulated. The NOD, LRR and pyrin domain-containing protein 3 (NLRP3) inflammasome is a cytosolic signaling complex that is mainly expressed in microglia and plays a role in their inflammatory responses [79]. Increased expression of NLRP3, caspase-1 and NF-κB along with positive immunostaining for IBA1 (marker of microglial activation) and GFAP (marker of astrocyte reactivity) were noted in the cortex of APP/PS1 mice. Several lines of evidence indicated that cGAS–STING detects cytosolic DNA and is activated by DNA damage and neuroinflammation [34,80]. γ-H2AX (marker for DNA damage) and cleaved caspase-3 (marker for apoptosis) showed a significant increase in the hippocampus and cortex of AD mouse brains. All these pathological changes were reversed by administering NAD+ precursor nicotinamide riboside (NR) [81]. Hence, compromised autophagy/mitophagy can lead to increased DNA release in the cytosol, thereby causing cGAS–STING activation, which further leads to neuroinflammation and cellular senescence.

One of the major functions of microglia is to maintain tissue homeostasis by engulfment and clearance of debris. The triggering receptor expressed on myeloid cells 2 (TREM2) is a cell surface receptor on the microglia, which plays a crucial role in the phagocytosis of various substrates such as Aβ, apoptotic neurons and bacteria [82,83,84]. Hence, TREM2 promotes clearance of Aβ and attenuates neuroinflammation by inhibiting the production of pro-inflammatory cytokines [85,86,87]. Furthermore, the anti-inflammatory effect of TREM2 is mediated by a change in microglial polarization from M1 (pro-inflammatory) to M2 (anti-inflammatory phenotype). Administration of recombinant cGAMP in a 5-month APP/PS1 mouse model of AD-restored memory functions, decreased Aβ plaques and neuronal death and ameliorated neuroinflammation. The stimulation of the cGAS–STING pathway induced the expression of triggering receptors expressed on myeloid cells 2 (TREM2), which decreased Aβ and neuronal loss. Furthermore, TREM2 induced microglia polarization from M1 towards M2 phenotype, thereby attenuating neuroinflammatory responses [88]. Lysophosphatidic acids, an oxidized form of low-density lipoproteins (LDLs), are bioactive signaling phospholipids that are implicated in the pathogenesis of AD [89]. Aberrant activation of LPA signaling has been shown to increase Aβ levels, phosphorylation of tau and neuritic retraction [90,91]. Lysophosphatidic acid treatment in BV2 murine microglial cells increased the protein expression of cGAS and STING. Furthermore, STING induced increased nuclear translocation of NFκB and IRF3 and transcription of several inflammatory genes. The results from these limited studies indicated that microglia play a crucial role in regulating cGAS–STING signaling and neuroinflammatory responses in AD.

**Figure 2:**
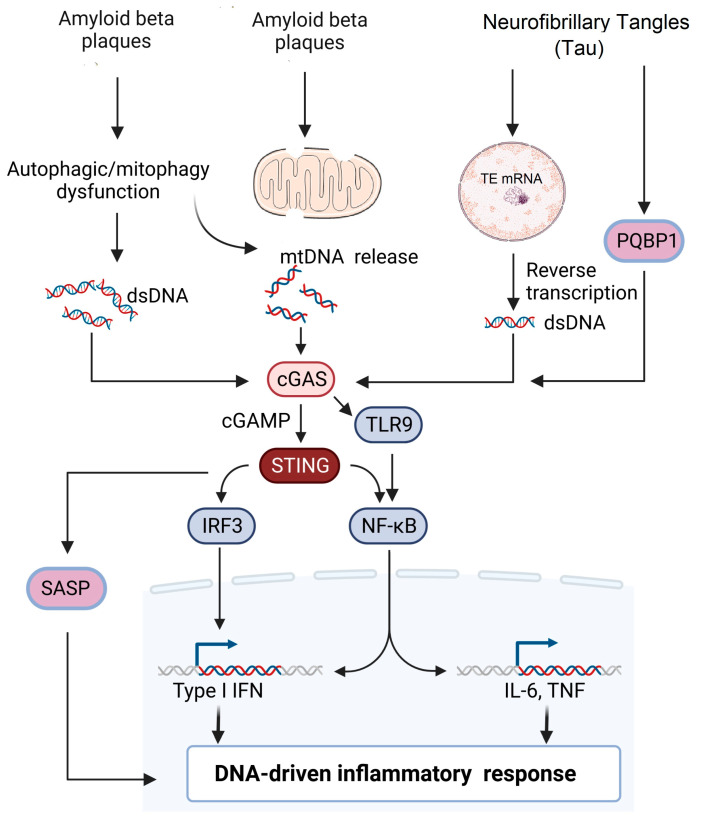
Potential signaling pathways involved in AD pathogenesis. Extracellular accumulation of β-amyloid plaques leads to either mitochondrial damage or impaired mitophagy leading to release of mitochondrial DNA into the cytosol. Impaired autophagy and cellular stress lead to accumulation of cytoplasmic dsDNA leading to the activation of the cGAS–STING pathway. Neurofibrillary tangles (NFT) can induce reverse transcription of transposable elements in the nucleus, leading to the accumulation of dsDNA. Alternatively, PGBP1 acts as an intracellular receptor for tau and activates the cGAS–STING pathway, which subsequently activates either the IRF3 or NF-κB pathway, leading to DNA-driven inflammatory response.

There are only limited studies that have evaluated the association between tau pathology and the cGAS–STING pathway. A recent study by Jin et al. showed that the tau protein interacts with Polyglutamine-binding protein 1 (PQBP1) and induces a neuroinflammatory response by activating the cGAS–STING pathway in microglial cells. Microglia-specific depletion of PQBP1 in primary cultures and PQPB1 knockout mice showed that PQPB1 is essential for sensing tau to induce nuclear translocation of NFκB and IRF3 (downstream transcription factors of the cGAS–STING pathway), increased transcription of inflammatory genes such as *Tnf and Isg54*. Finally, PQPB1 knockout mice injected with extracellular tau monomer showed less cognitive impairment. These results indicated that PQBP1 acted as an intracellular receptor for tau and activated the cGAS–STING pathway, specifically in the microglial cells [73]. The abnormal accumulation of tau protein has been shown to induce TE transcription as described earlier [92], and this unchecked TE expression leads to activation of cGAS–STING pathway, which leads to the activation of interferon regulatory factor 3 (IRF3), induction of type I interferons and activation of NF-κB [93]. More studies need to be performed to investigate how cGAS–STING regulates tau pathology in AD to determine therapeutic targets. The various mechanisms by which cGAS–STING pathway is involved in AD is summarized in Figure 2.

## 5. Current Therapeutics to Target STING in AD

Overstimulation of the cGAS–STING pathway and its associated neuroinflammation is thought to be associated with the development or progression of various neurodegenerative diseases, including AD. Hence, therapeutics inhibiting this pathway could attenuate disease progression in AD and other neurodegenerative diseases. The goal of developing agents that will inhibit the cGAS–STING pathway is possibly to protect neurons by alleviating chronic inflammation due to mitochondrial stress and autophagic dysregulation. On the other hand, inhibition of this pathway could increase susceptibility to infection that is secondary to the suppression of an innate immune response. Furthermore, the promotion of cancer can occur by limiting cellular senescence in cells with genomic instability/DNA damage. Although there are currently no cGAS–STING inhibitors currently approved for AD, studies using cell culture models and animal studies indicate that certain compounds are beneficial for attenuating the pathologies of AD (Table 1).

Before evaluating STING-based therapies in neuroinflammatory diseases like AD, several considerations must be addressed. First, there is not yet a consensus on which types of brain cell express cGAS and STING. Several studies have demonstrated conflicting evidence regarding the expression of cGAS and STING in different cell types [94,95,96]. For instance, activation of the STING–IFN cascade by cGAS–dsDNA interactions has been shown to mainly occur in the microglia and not the neurons or astrocytes [69,70]. Furthermore, cGAS is considered to be an IFN-stimulated gene (ISG); hence, it is likely that any cell with even low basal expression could upregulate this pathway in the disease contexts [97]. For instance, in a TBI model, the expression of STING and other ISGs were upregulated 2 months post-injury, while cGAS remained unaltered in comparison to sham levels [98]. Hence, evaluating the expression of cGAS and STING at baseline and during disease states over a period of time by using advanced techniques such as single-cell sequencing might be beneficial. Furthermore, current studies performed to determine the expression patterns of cGAS and STING are limited to rodents. Studies utilizing gyrencephalic animals (ferrets and non-human primates) and human samples need to be performed to validate the findings and study the expression patterns between species.

Second, considering the natural history of the disease (AD), the time course of cGAS and STING activation varies at different time points, and the subsequent window for treatment should be thoroughly characterized. In the context of AD, the main aim is to prevent inflammation and neuronal death. However, it is likely that there are critical times when cGAS–STING activation might be beneficial. Hence, a better understanding of the time of activation of cGAS or STING during the disease process will aid in providing therapeutic benefits.

Third, variability between species can hamper drug discovery and development because an unexpected species-specific role for STING as a receptor needs consideration. For instance, considerable molecular differences are noted between murine STING and human STING, and some agonists for murine STING are ineffective in activating human STING [99]. A study by Conlon et al. showed that DMXAA, a murine STING agonist, showed excellent antitumor activity in mouse models but in clinical trials it failed to bind to human STING because it acts specifically against murine STING. However, rat STING appears to display similar signaling profiles towards STING agonists when compared to human STING, suggesting that the rat is more suitable for the preclinical testing of STING-targeted therapies [100]. Using multiple animal models, including those having a gyrencephalic brain such as ferrets or non-human primates, may be beneficial for assessing cGAS–STING as a therapeutic target due to its species variability. Since none of the STING inhibitors has entered AD clinical trials, careful evaluation of blood–brain barrier permeability and side effects (infection and cancer induction) is needed [101].

## 6. Conclusions

The dysregulation of the innate immune cGAS–STING pathway and the associated neuroinflammation contribute to neurodegenerative diseases such as AD. Recent studies on cGAS–STING signaling in AD have increased our understanding of the role of this pathway in mediating neuroinflammation and neurodegeneration. Since the activation of cGAS–STING is context dependent in AD, the beneficial and detrimental effects need to be carefully evaluated while targeting this pathway. Furthermore, additional in-depth research needs to be performed to determine the temporal pattern of cGAS–STING activation at various time points in the course of AD pathogenesis to target this pathway for therapeutic benefit.

## Figures and Tables

**Table 1:** In vitro and in vivo studies evaluating cGAS–STING inhibitors in Alzheimer’s disease.

Model	Intervention	Outcome Measures	Results	References
5xFAD mice	H-151 (STING inhibitor)	-Neuroinflammatory genes (Hippocampus) -Aβ42 fractions (cortex) -Aβ (DG and cortex) -Iba1 and GFAP (DG) -Microglial phagocytosis activity	-Reduced -Reduced -Reduced -Reduced -Enhanced	[63]
-HMC3 human microglial cells -APP/PS1 mice	H-151 (STING inhibitor) NAD^+^ precursor nicotinamide riboside (NR)	- Aβ42 induced IL6 production -Neuroinflammatory markers [*Iba1 and GFAP,* *-Proinflammatory cytokines and chemokines, NLRP3,* *-γ-H2AX (DNA damage marker) and Cleaved-caspase-3 (apoptosis marker*] -cGAS and STING protein -Cellular senescence -Learning and Memory -Synaptic Plasticity (LTP)	-Inhibition -Reduced -Reduced -Reduced -Improved -Improved	[81]
-AANAT-KO mice (accelerated aging model) -AANAT-KO Primary cerebrocortical neurons	Melatonin Melatonin	- mtDNA release -cGAS/STING/IRF3 protein expression -Caspase-1 -Proinflammatory cytokines -MMP -Mitochondrial ROS -Cytosolic mtDNA -Caspase-1 -Proinflammatory cytokines	-Decreased -Decreased -Decreased -Decreased -Increased -Decreased -Decreased -Decreased -Decreased	[60]
-APP/PS1 mice	cGAMP	-Spatial memory - Aβ pathology -Proinflammatory cytokines -Neuronal apoptosis	-Improved -Reduced -Reduced -Reduced	[88]

## Data Availability

Not applicable.
